# Triglycerides/High-Density Lipoprotein Ratio and Coronary Artery Disease: Results from a Large Single-Center Study

**DOI:** 10.3390/jcm14041371

**Published:** 2025-02-19

**Authors:** Giuseppe De Luca, Matteo Nardin, Antonino Micari, Elvin Kedhi, Gennaro Galasso, Monica Verdoia, on behalf of the Novara Atherosclerosis Study Group (NAS)

**Affiliations:** 1 Department of Clinical and Experimental Medicine, Division of Cardiology, Policlinico G Martino, University of Messina, 98125 Messina, Italy; 2 Division of Cardiology, IRCCS Hospital Galeazzi-Sant’Ambrogio, 20157 Milan, Italy; 3 Department of Biomedical Sciences, Humanitas University, Pieve Emanuele, 20090 Milan, Italy; 4 Internal Medicine, Department of Medicine, ASST Spedali Civili, 25123 Brescia, Italy; 5 Department of Medicine, McGill University Health Center, Montreal, QC H4A 3J1, Canada; 6 Department Medical, University of Silesia, 40-032 Katowice, Poland; 7 Division of Cardiology, Ospedale Ruggi D’Aragona, Università di Salerno, 84084 Salerno, Italy; 8 Division of Cardiology, Ospedale degli Infermi, ASL Biella, 13875 Biella, Italy; 9 Department of Translational Medicine, Eastern Piedmont University, 28100 Novara, Italy

**Keywords:** TG/HDL, coronary atherosclerosis, triglycerides, HDL

## Abstract

**Background**. Despite the achievement of therapeutic goals regarding low-density lipoprotein cholesterol (LDL-C) levels with statins, high residual risk of events was reported in patients with coronary artery disease (CAD). Widespread attention has recently been focused on low plasmatic levels of high-density lipoproteins (HDLs) and high levels of triglycerides as risk factors for cardiovascular disease and as potential pharmacological targets, with particular attention paid to their ratio. Therefore, the aim of the current study was to investigate the association between triglycerides and HDLs and the TG/HDL ratio and their association with the prevalence and extent of CAD. **Methods**. We included patients undergoing non-urgent coronary angiography at Azienda Ospedaliera-Universitaria “Maggiore della Carità”, Novara, Italy, from 2007 to 2018. Patients chronically treated with triglyceride-lowering therapies (PUFA and Fibrates) were excluded from this analysis. Fasting samples were collected at the moment of angiography. CAD was defined as at least one vessel stenosis >50%. **Results**. Our study population of 5997 patients was divided according to TG/HDL ratio quartiles. The TG/HDL ratio was significantly associated with age, gender, smoking status, hypercholesterolemia, diabetes, and the chronic use of ACE inhibitors, statins, beta-blockers, aspirin, ADP antagonists, and diuretics. The TG/HDL ratio was additionally associated with several laboratory parameters. In multiple logistic regression analysis, HDLs but not the TG/HDL ratio were independently associated with the prevalence and extent of CAD. **Conclusions**. Our study showed that HDLs but not the TG/HDL ratio are independently associated with the extent and prevalence of CAD. Therefore, this ratio does not provide additional prognostic information to HDLs in the prediction of the prevalence and extent of this disease.

## 1. Introduction

Coronary artery disease (CAD) is a major determinant of mortality worldwide [1]. Despite the advancements in coronary revascularization, the advent of new technologies [2,3,4], and the development of antithrombotic therapies [5,6], outcomes remain unsatisfactory in both short- and long-term follow-ups for high-risk patient subsets [7,8]. Consequently, significant attention has been directed towards cardiovascular risk assessment over the last few decades, focusing on identifying new risk factors [8,9,10,11,12] and enhancing prevention strategies [13,14,15].

Dyslipidaemia is a major risk factor for CAD, particularly high levels of low-density lipoproteins (LDLs), which have been identified as the most atherogenic lipoproteins. Numerous studies have demonstrated the benefits of aggressively lowering LDL levels with high-dose statins in reducing atheromatous plaque progression and preventing cardiovascular events [16,17]. However, despite achieving therapeutic goals regarding LDL cholesterol (LDL-C) levels with statins, a high residual risk of events has been reported in patients with CAD [18,19].

Recently, attention has shifted to low plasma levels of high-density lipoproteins (HDLs) and high triglyceride levels as risk factors for cardiovascular disease and potential pharmacological targets [20,21,22,23,24,25,26,27], with a particular emphasis on their ratio. This ratio has been shown to reflect the degree of insulin resistance and is associated with a worse lipid profile, being inversely related to the size of both HDL and LDL particles, which are relevant factors in the atherosclerosis process.

However, controversy still exists regarding the role of the triglyceride/HDL (TG/HDL) ratio in CAD [28,29,30,31,32,33,34,35,36,37,38,39,40,41,42,43]. Therefore, the aim of the current study was to investigate the association between the TG/HDL ratio and the prevalence and extension of CAD.

## 2. Methods

We included a single-center cohort of patients (>18 years old) undergoing non-urgent coronary angiography at Azienda Ospedaliera-Universitaria, “Maggiore della Carità”, Novara, Italy, from 2007 to 2018. All demographic and clinical data were collected after obtaining written informed consent from the patient and included in a dedicated database. Patients chronically treated with triglyceride-lowering therapies (polyunsaturated fatty acids (PUFAs) and fibrates) were excluded from this analysis.

Hypertension was defined as systolic pressure > 140 mm Hg and/or diastolic pressure > 90 mm Hg or if the individual was taking antihypertensive medications. The diagnosis of diabetes was based on a previous history of diabetes treated with or without drug therapies, fasting glycaemia > 126 mg/dL, random glycaemia > 200 mg/dL, or HbA1c > 6.5%.

### 2.1. Biochemical Measurements

Blood samples were drawn following a fasting period of 12 h at the moment of angiography from patients undergoing elective or non-urgent coronary angiography. Glucose, creatinine, blood count, and lipid profile were determined by the standard methods.

### 2.2. Coronary Angiography

Coronary angiography was routinely performed, preferring the radial approach, using 6-French right and left heart catheters. Quantitative coronary angiography was performed by experienced interventional cardiologists using automatic edge-detection systems (Siemens Acom Quantcor QCA, Erlangen, Germany), as previously described [12].

Significant coronary artery disease was defined as at least 1 coronary stenosis more than 50%. Severe multivessel disease was defined as three-vessel disease and/or left main coronary artery disease. In the case of patients who had previously undergone percutaneous or surgical revascularization, the treated vessel was counted as significantly diseased.

### 2.3. Statistical Analysis

Statistical analysis was performed with the SPSS 23.0 statistical package. Continuous data were expressed as the median and interquartile range (IQR), and categorical data as percentages. Mann–Whitney and chi-square tests were used for continuous and categorical variables, respectively.

Multiple logistic regression analysis was performed to evaluate the independent relationship between TG/HDL ratio quartile values and coronary artery disease. Two separate models were built: the first including HDLs and the second additionally including all baseline confounding factors (biochemistry and clinical and demographic variables with a *p* value < 0.05) that were entered into the model in a block. A *p* value < 0.05 was considered statistically significant.

## 3. Results

**Figure 1:**
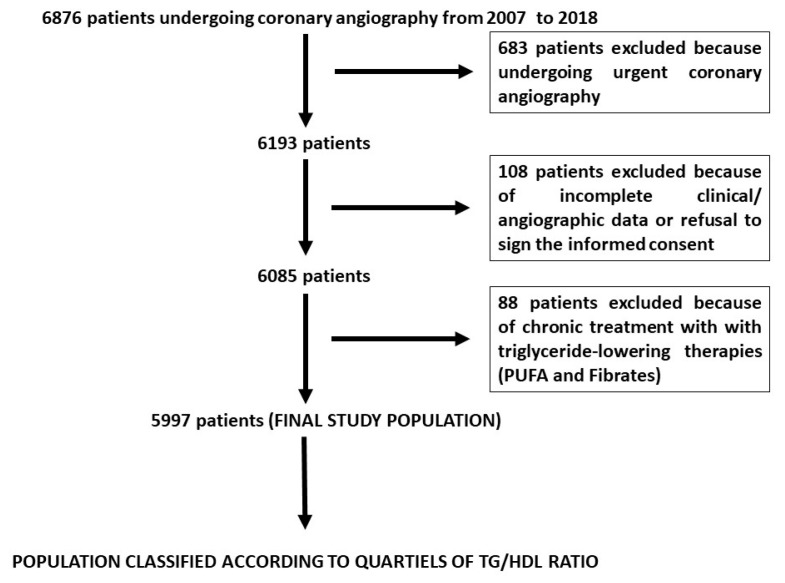
Study flowchart.

Our initial population was represented by 6876 patients undergoing coronary angiography from 2007 to 2018. A total of 5997 patients undergoing non-urgent coronary angiography represented our final study population (Figure 1), which was divided according to TG/HDL ratio quartiles. Patients’ demographic and clinical characteristics are presented in Table 1. The TG/HDL ratio was significantly associated with age (*p* < 0.001), gender (*p* < 0.001), body mass index (BMI) (*p* < 0.001), smoking status (*p* < 0.001), hypercholesterolemia (*p* < 0.001), diabetes (*p* < 0.001), metabolic syndrome (*p* < 0.001), family history of CAD (*p* = 0.003), previous myocardial infarction (MI) (*p* < 0.001), previous coronary artery bypass grafting (CABG) (*p* = 0.028), a previous cerebrovascular accident (CVA) (*p* = 0.028), and chronic kidney disease (*p* < 0.001). Concerning medical therapy at admission, the TG/HDL ratio was significantly associated with the use of angiotensin-converting enzyme (ACE) inhibitors (*p* = 0.004), statins (*p* = 0.004), nitrates (*p* = 0.011), beta-blockers (*p* < 0.001), acetylsalicylic acid (*p* < 0.001), ADP antagonists (*p* < 0.001), and diuretics (*p* = 0.046). The TG/HDL ratio was additionally associated with several laboratory parameters, such as platelet count (*p* = 0.028), white blood cell count (*p* < 0.001), hemoglobin (*p* < 0.001), glycemia (*p* < 0.001), glycosylated hemoglobin (*p* < 0.001), total cholesterol (*p* < 0.001), LDL cholesterol (*p* < 0.001), HDL cholesterol (*p* < 0.001), triglycerides (*p* < 0.001), and uric acid (*p* < 0.001).

**Figure 2:**
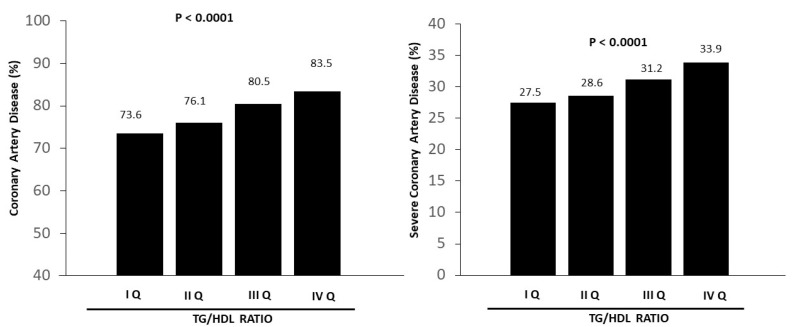
Bar graphs show the prevalence of coronary artery disease (**left graph**) and severe coronary artery disease (**right graph**) according to quartiles of the triglyceride/HDL ratio.

Angiographical features are shown in Table 2. A significant association was found between the TG/HDL ratio and the prevalence and extent of coronary artery disease (*p* < 0.001) (Figure 2), including disease of the left main coronary artery (*p* < 0.001), left anterior descending coronary (*p* < 0.001), circumflex coronary (*p* < 0.001), and right coronary (*p* < 0.001). We also identified a significant relationship with type C lesions (*p* < 0.001), coronary calcifications (*p* < 0.001), total chronic occlusion (*p* < 0.001), restenosis (*p* = 0.009), and TIMI flow (*p* < 0.001).

**Figure 3:**
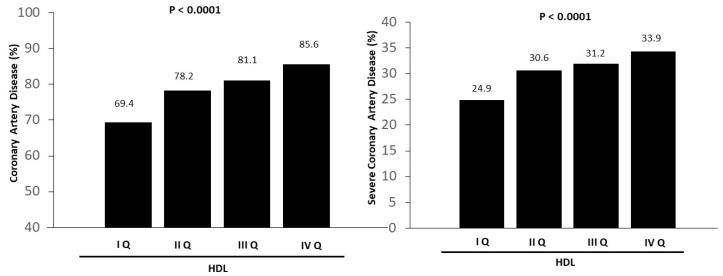
Bar graphs show the prevalence of coronary artery disease (**left graph**) and severe coronary artery disease (**right graph**) according to quartiles of HDL values.

The association between the TG/HDL ratio and the prevalence of CAD (OR [95% CI] = 1.22 (1.16–1.29), *p* < 0.001) disappeared in multiple logistic regression analysis after the inclusion of just HDL values (OR [95% CI] = 1.02 (0.96–1.10), *p* = 0.44). Similar results were observed in a second multivariate model after the inclusion of all baseline confounders (age, gender, BMI, smoking status, diabetes, hypercholesterolemia, metabolic syndrome, family history of CAD, previous MI, previous CABG, previous CVA, chronic use of ACE inhibitors, statins, beta-blockers, diuretics, nitrates, acetylsalicylic acid, or clopidogrel, platelet count, hemoglobin, WBC count, glycaemia, uric acid, fibrinogen, Hb1Ac, creatinine, and LDL cholesterol) (adjusted OR [95% CI] = 1.08 (0.99–1.19), *p* = 0.76). Similar results were observed for severe CAD (OR [95% CI] = 1.11 (1.05–1.17), *p* < 0.001; model 1, OR [95% CI] = 1.03 (0.97–1.10), *p* = 0.31; model 2, OR [95% CI] = 1.03 (0.95–1.11), *p* = 0.48). HDL was independently associated with the prevalence and extent of CAD in both multivariate models (Figure 3; Table 3).

## 4. Discussion

The main finding of this study is that the TG/HDL ratio does not provide additional prognostic information beyond HDL-C levels. Although the TG/HDL ratio is associated with CAD, this association disappears after adjusting for HDL-C alone and for other confounding factors.

CAD remains a major determinant of mortality and morbidity in developed countries. Over the past few decades, significant attention has been directed towards identifying risk factors for CAD and promoting its prevention. Several trials have demonstrated that aggressive LDL-C lowering can reduce the progression of atherosclerosis and cardiovascular events [13,14,15,16,17,18,19]. However, an important residual event rate was observed in most trials using statins, ranging from 9% to 20%, depending on the established target levels of LDL-C and statin dosage [13,16,17,18,20].

Over the last sixty years, HDL has been recognized as an independent modifier of the atherosclerotic process due to its cardioprotective role and potential to prevent coronary disease [21,22,23,24,25,26,27]. Although no conclusive studies have proven a reduction in event rates as a result of raising HDL-C [44,45,46,47], low concentrations of HDL-C have been proposed as a risk predictor.

In recent years, there has been significant interest in the TG/HDL ratio. However, controversy still exists regarding its independent association with the extent of CAD and its clinical outcomes. As observed in previous investigations, the combination of high triglyceride levels and low HDL-C levels predicts cardiovascular disease independent of LDL-C levels [28,29,30,31,32,33,41,43]. The TG/HDL ratio was found to be a reliable marker of metabolic syndrome and insulin resistance, both associated with endothelial damage and atherosclerosis [34,35]. Additionally, a high TG/HDL ratio, suggested as a sign of atherogenic dyslipidemia, has been linked to unfavorable outcomes at long-term follow-up in secondary prevention studies conducted on gender-specific groups with suspected coronary ischemic disease [34] and small country-specific high-risk patients [36], as well as in patients presenting with an acute coronary syndrome (ACS) [37].

In a recent large cohort of very high-risk patients with chronic coronary syndrome enrolled in an Italian registry, De Luca et al. [38] observed that patients in the highest tertile of the TG/HDL ratio were mainly males, significantly younger, and more likely to have risk factors such as diabetes mellitus, smoking, hypertension, chronic kidney disease, and higher levels of glycaemia, serum uric acid, total cholesterol, and LDL-C compared to patients in the lower tertiles. At 1-year follow-up, multivariable analysis showed that the TG/HDL ratio in this tertile did not emerge as an independent predictor of major adverse cardiocerebrovascular events (MACCE).

Similarly, in a recent study on ACS patients with STEMI [39], a high TG/HDL ratio did not impact 30-day and 1-year outcomes. The relatively short follow-up (1 year) may have potentially limited the conclusions of these studies, as a longer period may be needed to fully understand the prognostic impact of the TG/HDL ratio, as shown in previous studies [32,33,36,39]. Additionally, in the study by Darroudi et al. [40] including 1538 patients, the TyG index, but not the TG/HDL-C ratio, emerged as an independent marker for predicting the severity of coronary disease.

In our study, we analyzed a large consecutive cohort of patients undergoing non-urgent coronary angiography. We confirmed the important role of HDL-C in determining the extent of CAD. We found that the TG/HDL ratio was linearly associated with the prevalence and extent of CAD; however, it did not provide additional prognostic information compared to HDL-C alone. In line with previous negative studies on the association between this ratio and cardiovascular disease and outcomes, we observed that the association with CAD was no longer statistically significant after correcting for HDL-C alone or after including additional major confounding factors. In this final model, HDL-C maintained its independent association.

Several factors could explain the results of our study. While elevated triglyceride levels and a high TG/HDL ratio may be markers of insulin resistance, elevated triglyceride levels may have less impact on the atherosclerotic process compared to both LDL-C and especially HDL-C. In a recent prospective study including 300 patients with newly diagnosed significant (≥1 stenosis ≥ 50%) CAD undergoing computed tomography coronary angiography (CTCA), Oleksiak et al. [48] found that HDL-C, but not triglycerides, was independently associated with high-risk plaque features. Additionally, in a recent nationwide cohort study of about 5000 consecutive patients with chronic coronary syndrome with statin-controlled LDL-C levels [49], hypertriglyceridemia was present in around 24% of cases and did not predict MACCE at 1 year, supporting the conclusions of our study.

## 5. Limitations

The limitations of our study are those of any observational study, specifically not offering information about the long-term effects of the TG/HDL ratio. The use of intravascular imaging, such as the IVUS technique, would have improved the definition of CAD, especially in patients with complex eccentric plaques and with negative remodeling. Finally, we evaluated the extension and severity of coronary artery disease based on the number of disease vessels and involvement of the left main coronary artery, whereas the use of the Gensini [50] or Syntax score [51] would have further improved our results.

## 6. Conclusions

Our study showed that HDL-C but not the TG/HDL ratio is independently associated with the extent and prevalence of coronary artery disease. Therefore, this ratio does not provide additional prognostic information to HDL-C in the prediction of the prevalence and extent of this disease.

## Figures and Tables

**Table 1:** Clinical characteristics according to quartiles of the TG/HDL ratio.

Baseline Clinical Characteristics	I Quartile n = 1489	II Quartile n = 1502	III Quartile n = 1505	IV Quartile n = 1501	*p*-Value
Age (years, median [IQR])	72 (64–78)	71 (62–77)	69 (60–77)	66 (57–73)	<0.001
Age ≥ 75 years (n (%))	584 (39.2)	531 (35.4)	485 (32.2)	319 (21.3)	<0.001
Male sex (n (%))	552 (37.1)	460 (30.6)	443 (29.4)	417 (21.1)	<0.001
BMI (kg/m^2^, median [IQR])	25 (22.6–27.6)	26.1 (23.8–29)	26.8 (24.3–29.7)	27.7 (25.1–31.1)	<0.001
Hypercholesterolemia (n (%))	709 (47.6)	820 (54.6)	853 (56.7)	941 (62.7)	<0.001
Dyslipidemia (%)	709 (47.6)	832 (55.4)	956 (63.5)	1292 (86.1)	<0.001
Chronic kidney disease (n (%))	312 (21.0)	364 (24.2)	377 (25.0)	436 (29.0)	<0.001
Smokers (n (%))					<0.001
Active smokers	268 (18.0)	350 (21.5)	395 (26.2)	470 (31.3)	
Previous smoker	320 (23.3)	343 (22.8)	317 (21.1)	352 (23.5)	
Hypertension (n (%))	1068 (71.7)	1093 (72.8)	1084 (72.0)	1102 (73.4)	0.4
Diabetes mellitus (n (%))	422 (28.3)	499 (33.2)	589 (39.1)	708 (47.2)	<0.001
Metabolic syndrome (n (%))	313 (21.0)	434 (28.9)	583 (38.7)	771 (51.4)	<0.001
Previous MI (n (%))	302 (20.3)	319 (21.2)	342 (22.7)	406 (27.0)	<0.001
Previous PCI (n (%))	422 (28.3)	430 (28.6)	442 (29.4)	467 (31.1)	0.085
Previous CABG (n (%))	144 (9.7)	167 (11.1)	152 (10.1)	190 (12.7)	0.028
Previous CVA (n (%))	83 (5.6)	102 (6.8)	81 (5.4)	129 (8.6)	0.008
**Indication to angiography**					0.034
Stable Angina (n (%))	476 (32.0)	503 (33.5)	488 (32.4)	469 (31.2)	
ACS (n (%))	716 (48.1)	728 (48.5)	764 (50.8)	794 (52.9)	
DCM/Valvulopathy (n (%))	297 (19.9)	271 (18.0)	253 (16.8)	238 (15.9)	
**Concomitant medications**					
ACE inhibitors (n (%))	502 (33.7)	533 (35.5)	578 (38.4)	572 (38.1)	0.004
ARB (n (%))	317 (21.3)	338 (22.5)	317 (21.1)	363 (24.2)	0.13
Beta blockers (n (%))	742 (49.8)	800 (53.3)	864 (57.4)	849 (56.6)	<0.001
Nitrates (n (%))	460 (30.9)	498 (33.2)	546 (36.3)	518 (34.5)	0.011
Statins (n (%))	709 (47.6)	740 (49.3)	775 (51.5)	787 (52.4)	0.004
ASA (n (%))	814 (54.7)	901 (60.0)	912 (60.6)	925 (61.6)	<0.001
Clopidogrel (n (%))	267 (17.9)	334 (22.2)	341 (22.7)	348 (23.2)	0.001
Calcium channel blockers (n (%))	296 (19.9)	323 (21.5)	319 (21.2)	330 (22.0)	0.2
Diuretics (n (%))	455 (30.6)	467 (31.1)	515 (34.2)	497 (33.1)	0.046
**Biochemistry (median [IQR])**					
Platelets (10^3^/µL)	213 (174–252)	217 (181–260.75)	215 (180–259)	214 (180–256)	0.028
Hemoglobin (g/dL)	13.3 (12.2–14.4)	13.5 (12.2–14.6)	13.5 (12.2–14.7)	13.7 (12.3–14.8)	<0.001
WBC (10^3^/µL)	7.45 (6.06–9.10)	7.42 (6.20–9.07)	7.535 (6.20–9.1825)	7.91 (6.45–9.56)	<0.001
Glycaemia (mg/dL)	105 (93–123)	107 (95–126)	109 (96.25–129)	114 (100–151)	<0.001
HbA1c (%)	5.8 (5.5–6.3)	5.9 (5.5–6.4)	6.0 (5.6–6.7)	6.2 (5.7–7.0)	<0.001
Creatinine (mg/dL)	0.90 (0.75–1.08)	0.95 (0.79–1.15)	0.97 (0.80–1.17)	1.00 (0.85–1.23)	<0.001
HDL cholesterol (mg/dL)	52 (45–62)	42 (36–48)	37 (32–43)	32 (28–38)	<0.001
Total cholesterol (mg/dL)	162 (136–188)	154 (129–185)	157 (133–188)	163 (135–194)	<0.001
LDL cholesterol (mg/dL)	92 (71–115)	90 (69–117)	92 (71–118)	86 (64–113)	<0.001
Triglycerides (mg/dL)	71 (59.5–84.5)	99 (85–116)	130 (112–153)	204 (167–257)	<0.001
Uric acid (mg/dL)	5.3 (4.3–6.5)	5.7 (4.7–6.8)	6.0 (4.9–7.2)	6.4 (5.25–7.70)	<0.001
Fibrinogen (mg/dL)	369 (276–492)	388 (324–500)	401 (330–507)	395 (327–514)	<0.001

MI = myocardial infarction; PCI = percutaneous coronary interventions; CABG = coronary artery bypass grafting; ACS = acute coronary syndrome; DCM = dilated cardiomyopathy; ACE = angiotensin-converting enzyme; ARB = angiotensin receptor blockers; ASA = acetylsalicylic acid; WBC = white blood cells; LDL = low-density lipoprotein; HDL = high-density lipoprotein.

**Table 2:** Angiographic characteristics according to quartiles of the TG/HDL ratio.

Angiographic Features	I Quartile n = 3771	II Quartile n = 3785	III Quartile n = 3700	IV Quartile n = 3518	*p*-Value
CAD yes (n (%)) §	1096 (73.6)	1143 (76.1)	1212 (80.5)	1253 (83.5)	<0.001
Vessel disease					<0.001
1 (n (%)) §	446 (30.0)	411 (27.4)	416 (27.6)	447 (29.8)	
≥2 (n (%)) §	650 (43.7)	732 (48.7)	796 (52.9)	806 (53.7)	
Left main trivessel disease (n (%)) §	406 (27.5)	426 (28.6)	469 (31.2)	505 (33.9)	0.001
Left main coronary artery disease (n (%)) §	335 (8.9)	384 (10.1)	383 (10.4)	449 (12.8)	<0.001
LAD (n (%)) §	1981 (52.5)	2097 (55.4)	2195 (59.3)	2200 (62.5)	<0.001
CX (n (%)) §	1640 (43.5)	1695 (44.8)	1887 (51)	1864 (53)	<0.001
RCA (n (%)) §	1692 (44.9)	1813 (47.9)	1956 (52.9)	2040 (58)	<0.001
Type C lesion (n (%))	1005 (26.7)	996 (26.3)	1059 (28.6)	1079 (30.7)	<0.001
Lesion length (mm, median [IQR]))	20 (13–25)	20 (13–26)	18 (12–25)	20 (12–25)	0.007
Percent stenosis (%, median [IQR]))	90 (80–99)	85 (80–99)	90 (80–99)	90 (80–99)	0.072
Reference diameter (mm, median [IQR]))	3 (2.5–3.38)	3 (2.5–3.25)	3 (2.5–3.5)	3 (2.5–3.5)	0.69
Calcifications (n (%))	447 (11.9)	467 (12.3)	503 (13.6)	546 (15.5)	<0.001
Chronic occlusion (n (%))	372 (9.9)	401 (10.6)	444 (12.0)	472 (13.4)	<0.001
Restenosis (n (%))	139 (3.7)	128 (3.4)	157 (4.2)	164 (4.7)	0.009
Thrombus (n (%))	133 (5.5)	128 (4.9)	134 (4.9)	131 (4.8)	0.88
Bifurcation lesion (n (%))	575 (15.2)	549 (14.5)	587 (15.9)	556 (15.8)	0.26
TIMI Flow					<0.001
3	3119 (82.7)	3112 (82.2)	2966 (80.2)	2766 (78.6)	
2	112 (3.0)	128 (3.4)	133 (3.6)	96 (2.7)	
1	67 (1.8)	72 (1.9)	78 (2.1)	74 (2.1)	
0	473 (12.5)	473 (12.5)	523 (14.1)	582 (16.5)	

§ Per patient definition. CAD = coronary artery disease; LAD = left anterior descending; CX = circumflex coronary artery; RCA = right coronary artery; TIMI = thrombolysis in myocardial infarction.

**Table 3:** Multivariate logistic regression analysis.

	Adjusted OR	95% CI	*p* Value
**CAD**			
**MODEL 1**			
TG/HDL Ratio (quartiles)	1.02	0.96–1.1	0.44
HDL (quartiles)	0.74	0.69–0.8	<0.001
MODEL 2 *			
TG/HDL Ratio (quartiles)	1.08	0.99–1.19	0.76
HDL (quartiles)	0.84	0.76–0.92	<0.001
**Severe CAD**			
**MODEL 1**			
TG/HDL Ratio (quartiles)	1.03	0.97–1.10	0.31
HDL (quartiles)	0.89	0.83–0.95	<0.001
MODEL 2 *			
TG/HDL Ratio (quartiles)	1.03	0.95–1.11	0.48
HDL (quartiles)	0.93	0.86–0.99	0.048

OR = odds ratio; CI = confidence interval; CAD = coronary artery disease; TG = triglyceride; HDL = high-density lipoprotein. * Variables included in the model: age, gender, smoking status, diabetes, hypercholesterolemia, family history of CAD, previous MI, previous CABG, previous CVA, chronic use of ACE inhibitors, statins, beta-blockers, diuretics, nitrates, acetylsalicylic acid, or clopidogrel, platelet count, hemoglobin, WBC count, glycaemia, uric acid, fibrinogen, Hb1Ac, creatinine, LDL cholesterol, BMI.

## Data Availability

Data are available upon specific request.
